# Evaluating Intensive Insulin Therapy With Empagliflozin in Type 2 Diabetes: A Randomised Study

**DOI:** 10.1002/edm2.70096

**Published:** 2025-08-19

**Authors:** Nobutoshi Fushimi, Hiroki Hachiya, Tatsuya Iwasaka, Machi Nagao, Tomoki Masamura, Kohei Higashi, Akihiro Mori

**Affiliations:** ^1^ Department of Endocrinology and Diabetes Ichinomiyanishi Hospital Ichinomiya Japan

**Keywords:** glucotoxicity, hyperglycemia, intensive insulin therapy, SGL2 inhibitor

## Abstract

**Aims/Introduction:**

Glucotoxicity exacerbates hyperglycemia by impairing insulin secretion and sensitivity, necessitating effective interventions. Although short‐term intensive insulin therapy (SIIT) mitigates glucotoxicity, the effect of combining SIIT with sodium‐glucose co‐transporter 2 (SGLT2) inhibitors in hospitalised type 2 diabetes mellitus (T2DM) patients with severe hyperglycemia remains unclear. Herein, we aimed to evaluate the efficacy and safety of combining SGLT2 inhibitors with basal bolus therapy (BBT) for glycemic control in hospitalised patients with T2DM.

**Materials and Methods:**

In this randomised, open‐label, single‐centre trial, 35 eligible T2DM patients hospitalised for treating hyperglycemia were allocated to the BBT (*n* = 17) or BBT with empagliflozin (BBT + E) groups (*n* = 18). Patients were monitored for 7 days using flash glucose monitoring. The primary outcome was time‐in‐range (TIR, 70–180 mg/dL). The secondary outcomes included time‐above‐range (TAR), time‐below‐range (TBR), daily glucose levels, total daily insulin dose and ketone body concentration.

**Results:**

The BBT + E group exhibited a significantly higher TIR from day 2, which exceeded 70% by day 5, with reduced TAR and insulin requirements. Blood glucose levels declined more rapidly in the BBT + E group, accompanied by a modest ketone elevation without severe ketoacidosis. The TBR increased marginally on day 7, primarily nocturnally; but no symptomatic hypoglycaemia occurred.

**Conclusion:**

The addition of SGLT2 inhibitors to BBT significantly improved early glycaemic control and reduced insulin requirements without severe ketone elevation in hospitalised T2DM patients. Routine monitoring of ketone levels and careful insulin titration are critical to ensure safety.

## Graphical abstract

This study compared the efficacy of intensive insulin therapy with empagliflozin (BBT + E) versus intensive insulin therapy alone (BBT) in hospitalised patients with type 2 diabetes admitted for hyperglycemia. The BBT + E group achieved an earlier improvement in time‐in‐range (TIR) compared to the BBT group. Although a mild increase in ketone bodies was observed, it remained within clinically acceptable levels.

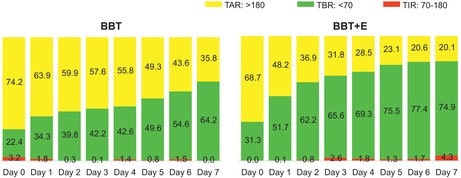

## 1 Introduction

Hyperglycemia can become exacerbated by the worsening of existing hypoinsulinemia and peripheral insulin resistance, thereby creating a vicious cycle known as glucotoxicity [1]. To address this issue, short‐term intensive insulin therapy (SIIT) has historically been used in patients with type 2 diabetes mellitus (T2DM) experiencing severe hyperglycemia [2, 3, 4, 5]. This approach aims to achieve early glycemic control, mitigate glucotoxicity, restore beta‐cell function and improve insulin sensitivity.

Although conventional oral antidiabetic agents often target insulin secretion and sensitivity, their efficacy in mitigating glucotoxicity is limited. Even incretin‐based therapies, which can enhance insulin secretion, have shown diminished effectiveness due to the decreased expression of incretin receptors under conditions of glucotoxicity [6, 7]. Thus, the ability of incretin‐related drugs to reverse glucotoxicity remains unclear.

In contrast, relatively novel sodium‐glucose co‐transporter 2 (SGLT2) inhibitors lower blood glucose levels through a unique mechanism of action by inhibiting SGLT2 activity in the proximal renal tubules, thereby promoting urinary glucose excretion independent of insulin activity. Clinically, SGLT2 inhibitors have demonstrated the potential to rapidly improve hyperglycemia and reduce the required insulin dose, particularly in patients with treatment‐resistant hyperglycemia [8]. However, increased fatty acid metabolism and ketone body synthesis induced by SGLT2 inhibitors raise concerns about the risk of ketosis and ketoacidosis (DKA) [9, 10, 11, 12], particularly in patients with compromised beta‐cell function and inadequate insulin secretion.

Therefore, this study aimed to evaluate the efficacy and safety of combining SGLT2 inhibitors with conventional intensive insulin therapy in hospitalised patients with T2DM who presented with severe hyperglycaemia.

## 2 Materials and Methods

### 2.1 Study Design and Participants

This was a prospective, randomised, open‐label, single‐centre trial. The participants included patients with T2DM who were hospitalised to general wards from the outpatient setting to alleviate glucotoxicity and improve BG control, with no prior history of SGLT2 inhibitor use. Eligible participants were aged 20 years or older, with a BMI of at least 18 kg/m^2^. Inclusion criteria varied based on prior treatment: for untreated patients or those on diet therapy only, an HbA1c ≥ 10%; for those on oral antidiabetic drugs or once‐daily insulin injections, an HbA1c ≥ 8.0% was required. Exclusion criteria included type 1 diabetes, multiple daily injection therapy, pregnancy, weekly use of DPP‐4 inhibitors or glucagon‐like peptide 1 (GLP‐1) receptor agonists or dual GLP‐1 and glucose‐dependent insulinotropic peptide receptor agonists, a history of DKA or hyperglycaemic coma, prednisone ≥ 10 mg/day, cancer, ICU admission, severe infections, advanced retinopathy, eGFR < 30 mL/min/1.73 m^2^, liver cirrhosis (Child‐Pugh B or higher), history of gastrointestinal operation or planned surgery during the study period. To exclude infections and stress‐related hyperglycaemia, all participants underwent chest radiography, general biochemical tests including C‐reactive protein and a complete blood count, and abdominal computed tomography upon hospital admission.

The study protocol was registered with the University Hospital Medical Information Network Clinical Trials Registry (UMIN000053224) and approved by the Ethics Committee of Ichinomiya Nishi Hospital. All participants provided informed consent.

### 2.2 Procedures

Baseline blood samples, including blood ketone bodies, were collected within one week prior to hospital admission to screen for ketosis, DKA, anti‐GAD antibodies and concomitant infections. After admission, all oral antidiabetic medications were discontinued. Participants were stratified into four groups based on the presence or absence of insulin injection use and HbA1c levels (> 10% or 8%–10%). Randomisation was conducted using block randomisation with a block size of four within either the intensive insulin therapy alone group (BBT group) or the intensive insulin therapy combined with empagliflozin 10 mg/day (BBT + E group). The study period spanned from the day of admission (day 0) until day 7 thereafter. On day 0, all patients were equipped with the FreeStyle Libre Pro (Abbott Japan Diabetes Care Inc., Tokyo, Japan). Basal bolus therapy (BBT) was initiated in both groups from day 0, while the BBT + E group began empagliflozin administration on day 1. During the treatment period, flash glucose monitoring (FGM) was conducted continuously and BG was measured before meals and at bedtime. Urine ketone tests were performed every morning, and blood ketone levels were measured on days 1, 3 and 7 before breakfast, using the same POC testing device as for BG measurements. Both blood glucose and blood ketone levels were measured using point‐of‐care (POC) metres (Niprostat Strip XP3, Nova Biomedical, Waltham, MA, USA). Meals were standardised with total daily calories calculated as ideal body weight (height × height × 22) × 30 kcal. Macronutrient composition was set at 60% carbohydrates, 18% protein and 22% fat [13]. Regarding insulin administration, we initiated treatment with a low dose and set a relatively strict target fasting blood glucose level of 95–120 mg/dL, based on previous studies on glycaemic control in non‐ICU hospitalised patients with type 2 diabetes [14, 15, 16, 17]. Basal insulin (glargine U‐300) was initiated at a dose of 0.15 U/kg/day (maximum 10 units/day) for patients under 70 years old and 0.10 U/kg/day (minimum 4 units/day, maximum 8 units/day) for those aged 70 years or older. Dose adjustments were made daily based on fasting blood glucose (FBG) levels: no change for 95–120 mg/dL, a 10% increase for 121–140 mg/dL and a 20% increase for ≥ 141 mg/dL. Dose reductions of 10%, 20% or 30%–40% were applied for FBG levels of 70–94 mg/dL, 40–70 mg/dL or < 40 mg/dL, respectively. Bolus insulin (Lispro), divided into three equal doses of basal insulin, was administered before each meal. Sliding scale insulin was not used in this study. Hypoglycaemia was defined as the presence of hypoglycaemia or a BG level < 70 mg/dL. For hypoglycaemic episodes, the patients received 10 g of oral glucose until resolution.

Treatment failure was defined as severe hypoglycemia (BG < 40 mg/dL) occurring twice or more, a pre‐meal BG level > 400 mg/dL, ketosis (defined as urinary ketone level of ≥ 3 + or β‐OHB ≥ 1.0 mmol/L) or DKA (defined as β‐OHB level ≥ 3.0 mmol/L). Severe hypoglycemia was managed with intravenous glucose supplementation, whereas hyperglycemia, ketosis and DKA were treated with intensive continuous intravenous insulin therapy.

### 2.3 Outcomes

The primary outcome was the time‐in‐range (TIR; 70–180 mg/dL) measured daily using FGM. Secondary outcomes included time‐above‐range (TAR; 181–250 mg/dL), time‐below‐range (TBR; level 1 < 70 mg/dL, level 2 < 54 mg/dL), pre‐meal and bedtime BG levels measured by POC metres, β‐hydroxybutyrate (β‐OHB) concentrations by POC metres, urine ketone tests and total daily insulin dose (TDD).

### 2.4 Analysis

This exploratory pilot study was designed with a significance level of 5% and a statistical power of 80%, considering feasibility constraints and the limited number of eligible cases. The sample size calculation was based on the results of a previous study, assuming a mean difference of 27 mg/dL in blood glucose level changes between the two groups and a standard deviation of 27 mg/dL [18]. Accordingly, a total of 32 participants (16 per group) were required, and the target sample size was set at 36 participants to account for potential dropouts. Data analysis was performed using EZR software (Saitama Medical Center, Jichi Medical University, Saitama, Japan) [19]. All available data during hospitalisation were included in the analysis. Continuous variables following a normal distribution were reported as mean ± standard deviation and analysed utilising the t‐test, while for those not following a normal distribution, data were expressed as medians and interquartile ranges and analysed using the rank‐sum test. Non‐continuous variables are presented as absolute numbers and percentages. Urinary ketone test results were transformed to an ordinal scale and subsequently analysed using the Mann–Whitney U test. Other categorical variables were analysed using the chi‐square test.

## 3 Results

**FIGURE 1:**
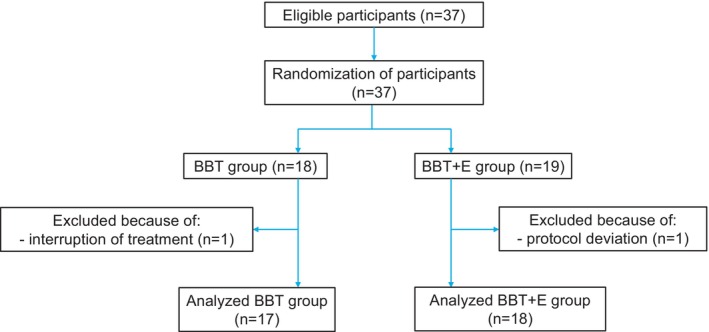
Patient disposition flowchart. BBT, basal bolus therapy; BBT + E, BBT plus empagliflozin.

A total of 37 eligible T2DM patients were admitted to our hospital between 26 December2023, and 1 August 2024. The patients were recruited and randomly allocated into stratified groups. Two patients were excluded from the analysis, one due to treatment interruption immediately after admission and the other due to protocol deviation. Consequently, data from 35 patients were analysed: 17 in the BBT group and 18 in the BBT + E group (Figure 1). The baseline characteristics of the patients are summarised in Table 1. No significant differences were observed between the two groups in terms of age, sex, BMI, medical history, HbA1c, BG, C‐peptide reactivity, β‐OHB by a central laboratory reference method, estimated glomerular filtration rate (eGFR), microvascular and macrovascular complications, previous treatments, or diabetes medications.

**TABLE 1:** Clinical characteristics of study patients.

	BBT	BBT + E	P‐value
Number of patients	17	18	
Age	65.6 ± 16.8	63.8 ± 15.2	0.748
Male, *n* (%)	11 (65)	7 (39)	0.181
Body mass index, kg/m^2^	27.4 ± 5.8	24.5 ± 4.2	0.111
Duration of diabetes, years	10.8 ± 13.5	7.9 ± 9.5	0.487
HbA1c, %	11.1 ± 2.1	11.2 ± 1.8	0.975
Glu, mg/dl	251.7 ± 69.3	269.8 ± 101.8	0.543
C‐peptide reactivity, ng/dl	3.0 ± 1.2	3.0 ± 1.0	0.901
β‐OHB, mmol/L	0.05 [0.04, 0.17]	0.09 [0.02, 0.20]	0.759
eGFR, mL/min/1.73m^2^	69.5 ± 22.35	69.3 ± 27.7	0.986
Microvascular complications			
Diabetic nephropathy (%)	9 (53)	7 (39)	0.505
Diabetic retinopathy (%)	3 (18)	6 (33)	0.443
Diabetic neuropathy (%)	9 (53)	8 (44)	0.740
≥ 2 microvascular complication (%)	8 (47)	7 (39)	0.738
Macrovascular disease			
Coronary artery disease (%)	2 (12)	2 (11)	1.000
Cerebrovascular disease (%)	2 (12)	2 (11)	1.000
Peripheral arterial disease (%)	0 (0)	1 (5.6)	1.000
≥ 1 macrovascular condition (%)	4 (24)	4 (22)	1.000
Previous treatment			
Diet alone, *n* (%)	6 (36)	8 (44)	0.733
Oral agents only, *n* (%)	8 (47)	7 (39)	0.738
Daily GLP‐1RA + oral agents, *n* (%)	0 (0)	1 (5.6)	1
Insulin+oral agents, *n* (%)	2 (11.8)	2 (11.1)	1
Insulin+Daily GLP‐RA + oral agents, *n* (%)	1 (5.9)	0 (0)	0.486
Drug agents			
DPP4 inhibitor, *n* (%)	10 (59)	8 (44)	0.505
Biguanide, *n* (%)	8 (47)	4 (22)	0.164
Sulfonylurea, *n* (%)	3 (18)	2 (11)	0.658
Alpha‐glucosidase inhibitor, *n* (%)	0 (0)	1 (6)	1
Thiazolidine, *n* (%)	2 (12)	0 (0)	0.229
Basal insulin, *n* (%)	3 (18)	2 (11)	0.658
Daily GLP‐1RA, *n* (%)	1 (6)	1 (6)	1

Abbreviations: β‐OHB, β‐hydroxybutyrate; eGFR, estimated glomerular filtration rate. DPP‐4, dipeptidyl peptidase‐4; GLP‐1RA, glucagon‐like peptide 1 receptor agonists.

**FIGURE 2:**
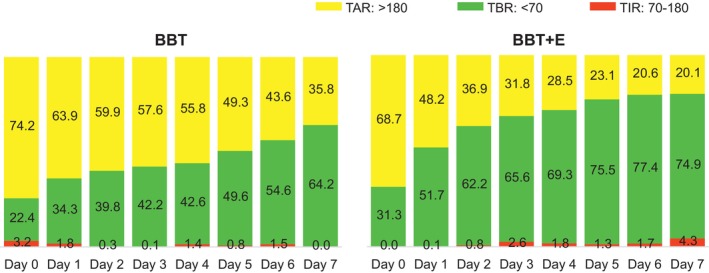
Daily FGM metrics. FGM, flash glucose monitoring; BBT, basal bolus therapy; BBT + E, BBT plus empagliflozin; TAR, time‐above‐range; TIR, time‐in‐range; TBR, time‐below‐range.

Table 2 summarises the study outcomes of FGM and ketone body. The TIR was significantly higher in the BBT + E group starting from day 2 of treatment and remained elevated until day 6. Conversely, the TAR was significantly lower in the BBT + E group from day 2 and persisted until day 7. TBR at blood glucose levels < 70 mg/dL was significantly higher in the BBT + E group on day 7, although TBR at levels < 54 mg/dL was rare in both groups (Figure 2).

**TABLE 2:** Study outcomes of FGM and ketone body.

	Day 0			Day 1			Day 2			Day 3		
	BBT (*n* = 17)	BBT + E (*n* = 18)	*P*	BBT (*n* = 17)	BBT + E (*n* = 18)	*P*	BBT (*n* = 17)	BBT + E (*n* = 18)	*P*	BBT (*n* = 17)	BBT + E (*n* = 18)	*P*
TAR: > 180, %	74.2 ± 35.1	68.7 ± 27.4	0.634	63.9 ± 27.3	48.2 ± 29.0	0.132	59.9 ± 28.4	36.9 ± 32.3	0.044*	57.6 ± 30.1	31.8 ± 22.7	0.012*
TIR: 70–180, %	22.4 ± 29.7	31.3 ± 27.4	0.394	34.3 ± 27.4	51.7 ± 28.8	0.096	39.8 ± 28.4	62.2 ± 31.5	0.047*	42.2 ± 29.9	65.6 ± 20.5	0.017*
TBR level 1: < 70, %	3.2 ± 12.9	0	0.341	1.8 ± 6.4	0.1 ± 0.5	0.326	0.3 ± 1.3	0.8 ± 2.1	0.487	0.1 ± 0.4	2.6 ± 7.6	0.210
TBR level 2: < 54, %	2.3 ± 9.1	0	0.341	1.3 ± 4.5	0	0.289	0	0	—	0	0.2 ± 0.8	0.310
β‐OHB, mmol/L				0.1 [0.1–0.2]	0.2 [0.1–0.3]	0.262				0.2 [0.1–0.3]	0.5 [0.2–0.6]	0.021*
Urine ketone ‐, *n* (%)	15 (94)	18 (100)	0.316	16 (100)	17 (94)	0.377	15 (94)	14 (82)	0.374	14 (88)	15 (88)	1.000
+, *n* (%)	1 (6)	0		0	1 (6)		0	2 (12)		1 (6)	0	
2+, *n* (%)	0	0		0	0		1 (6)	1 (6)		1 (6)	2 (12)	
3+, *n* (%)	0	0		0	0		0	0		0	0	
TDD, U/day	8.6 ± 2.7	8.4 ± 3.3	0.890	16.6 ± 4.1	15.9 ± 4.7	0.614	19.1 ± 5.1	18.0 ± 5.5	0.538	21.6 ± 6.2	19.6 ± 5.9	0.330
	Day 4			Day 5			Day 6			Day 7		
	BBT (*n* = 17)	BBT + E (*n* = 18)	*P*	BBT (*n* = 17)	BBT + E (*n* = 18)	*P*	BBT (*n* = 17)	BBT + E (*n* = 17)	*P*	BBT (*n* = 16)	BBT + E (*n* = 16)	*P*
TAR: > 180, %	55.8 ± 28.2	28.5 ± 15.0	0.024*	49.3 ± 24.6	23.1 ± 18.4	0.023*	43.6 ± 22.8	20.6 ± 20.1	0.007*	35.8 ± 16.9	20.1 ± 18.0	0.023*
TIR: 70–180, %	42.6 ± 25.6	69.3 ± 14.3	0.013*	49.6 ± 23.5	75.5 ± 18.5	0.021*	54.6 ± 25.3	77.4 ± 20.8	0.012*	64.2 ± 16.9	74.9 ± 17.1	0.101
TBR level 1: < 70, %	1.4 ± 4.9	1.8 ± 4.4	0.827	0.8 ± 3.1	1.3 ± 5.1	—	1.5 ± 5.6	1.7 ± 4.7	0.913	0	4.3 ± 8.0	0.046
TBR level 2: < 54, %	0	0	—	0	0	—	0	0	—	0	0	—
β‐OHB, mmol/L										0.1 [0.1–0.3]	0.3 [0.2–0.4]	0.049*
Urine ketone ‐, *n* (%)	14 (88)	15 (88)	0.975	15 (94)	15 (88)	0.588	16 (100)	12 (75)	0.039*	16 (100)	13 (87)	0.150
+, *n* (%)	2 (13)	0		1 (6)	1 (6)		0	3 (19)		0	2 (13)	
2+, *n* (%)	0	2 (12)		0	1 (6)		0	1 (6)		0	0	
3+, *n* (%)	0	0		0	0		0	0		0	0	
TDD, U/day	23.5 ± 7.1	20.4 ± 6.4	0.188	25.3 ± 8.2	20.7 ± 6.5	0.084	28.6 ± 12.8	22.2 ± 7.3	0.089	31.8 ± 0.7	22.4 ± 0.3	0.029*

Abbreviations: TAR, time‐above‐range; TIR, time‐in‐range; TBR, time‐below‐range. β‐OHB, β‐hydroxybutyrate; TDD, total daily insulin dose.

**FIGURE 3:**
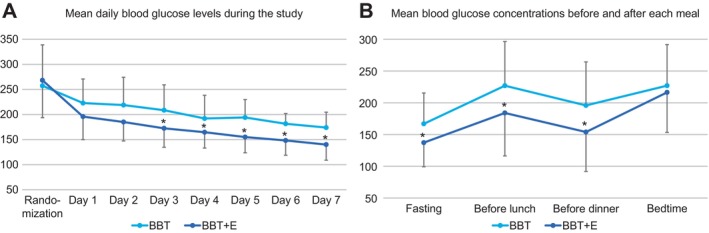
Differences in glycemic control in hospitalised patients with type 2 diabetes mellitus treated with BBT and BBT + E. (A) Mean blood glucose concentrations before and after each meal. (B) Mean daily blood glucose levels during the study. BBT, basal bolus therapy; BBT + E, BBT plus empagliflozin.

Fasting β‐OHB levels by POC metres were significantly higher in the BBT + E group on days 3 and 7, with the percentage of urinary ketones showing a slight increase on day 6. The TDD of insulin tended to be lower in the BBT + E group from day 3 onwards and reached statistical significance on day 7. Moreover, BG levels measured using POC glucometers demonstrated a significant reduction in the mean daily BG levels in the BBT + E group starting from day 3. This reduction was also evident in mean pre‐meal BG levels (Figure 3).

No symptoms of urinary tract or genital infections were observed in either group. Severe hypoglycaemia with BG levels below 40 mg/dL was not observed in either group. Two patients in the BBT group experienced one episode each of hypoglycaemia requiring glucose administration, and one such episode occurred in a single patient in the BBT + E group. All episodes were promptly resolved with glucose administration. Furthermore, no cases of DKA or preprandial BG levels exceeding 400 mg/dL were observed in either group, and no participants discontinued the study.

## 4 Discussion

This study demonstrated that in the management of blood glucose in hospitalised T2DM patients with hyperglycemia, combination therapy with BBT and empagliflozin achieved considerably higher TIR and lower insulin requirements at an earlier stage than conventional BBT alone.

The recommended TIR target for T2DM outpatients using FGM is 70%. However, no specific TIR target has been established for T2DM inpatients. Previous studies on SIIT aimed at FBG < 110 mg/dL and postprandial BG < 144 mg/dL to resolve glucotoxicity [3, 20, 21]. Recent literature indicates that implementing an 8‐point capillary BG measurement during SIIT and utilising stricter target ranges for BG levels—70–108 mg/dL for preprandial BG and 70–140 mg/dL for postprandial BG 2 h after meals—demonstrated beneficial effects in restoring beta‐cell function and insulin sensitivity when the target achievement rate was 65% or higher [22]. Furthermore, regarding the duration until the resolution of glucotoxicity, when clinical resolution is defined as the point at which TDD begins to decrease, SIIT using subcutaneous injections has been reported to require an average of 8.9 ± 4.3 days [23].

In this study, the BBT + E group demonstrated a significant increase in TIR by day 2, exceeding 70% on day 5, and this level was maintained thereafter. The TDD in the BBT + E group showed a slight increase after day 4 and remained lower than that in the BBT group. The rapid onset of the hypoglycemic effects of SGLT2 inhibitors [24] likely contributes to the early increase in TIR and resolution of glucotoxicity. In contrast, the TIR in the BBT group increased more gradually, reaching 65% by day 7, with the TDD continuing to increase throughout the study period, indicating incomplete glucotoxicity resolution.

The insulin dose progressively increases following the initiation of SIIT, and after glucotoxicity resolves, the insulin dose turns to decrease [23]. However, failure to appropriately reduce the insulin dosage leads to an increased risk of hypoglycemia [24]. The BBT + E group demonstrated a narrower range of insulin dose adjustments without dose reduction, which potentially minimised the risk of hypoglycemia. Previous studies have shown that combining insulin therapy with SGLT2 inhibitors in outpatients does not increase the risk [25, 26] and may reduce nocturnal hypoglycemia [27]. In the current study, the increased TIR was attributed to reductions in TAR without increases in TBR until day 6. However, on day 7, the BBT + E group exhibited a significant increase in TBR level 1, which was mainly observed during the nocturnal period. The low frequency of hypoglycemia observed in the BBT group may be attributed to its inability to resolve glucotoxicity. The FGM readings predominantly fell within the 60–69 mg/dL range, and no hypoglycemic symptoms were reported. Therefore, the possibility of pseudohypoglycemia cannot be excluded because of the significant margin of error associated with FGM measurements in the hypoglycemic range [28]. Additionally, the fact that SGLT2 inhibitors do not exert effects in the hypoglycemic range suggests that insulin administration may play a primary role in the occurrence of hypoglycemia. Further investigations are warranted to establish appropriate protocols for insulin administration in patients receiving SGLT2 inhibitors in the BBT group.

Regarding ketone dynamics, this study observed significant increases in blood and urine ketones with SGLT2 inhibitor treatment, which is consistent with previous reports. Elevated ketone levels are frequently observed during treatment with SGLT2 inhibitors, with fasting ketone concentrations reportedly increasing by approximately twofold [29]. In a previous study, administration of empagliflozin at a dosage of 25 mg/day for 28 days in T2DM patients who exhibited relatively well‐controlled glycemia resulted in β‐OHB levels ranging from 0.24 mmol/L to 0.56 mmol/L. [30] These findings are consistent with our results and reflect an increase within a clinically safe range. These small increases in ketone bodies may exert anti‐inflammatory, antioxidant and cardiorenal protective effects, potentially offering additional benefits [29]. However, DKA associated with SGLT2 inhibitors can occasionally present as a severe condition [31] that develops shortly after initiation of treatment [32]. SGLT2 inhibitor‐induced DKA often manifests as normoglycemic acidosis, which may not be promptly detected if blood glucose levels are monitored alone. Therefore, routine monitoring of urinary and blood ketone levels is essential for early detection and appropriate management [33]. Patients with type 1 diabetes mellitus, ketosis, DKA or acute complications were excluded, and insulin therapy was initiated on day 0, the day before SGLT2 inhibitor administration. This protocol may enhance therapeutic safety.

The efficacy of combining insulin therapy with SGLT2 inhibitors for glycemic management in outpatients has been previously reported [34, 35]. The results of this study suggest that the addition of SGLT2 inhibitors to BBT may also offer a relatively safe and effective approach to achieving target blood glucose levels in hospitalised patients with type 2 diabetes mellitus and hyperglycaemia. This strategy may contribute to shortening the overall treatment duration and reducing the length of hospital stay. To examine this possibility, we retrospectively analysed hospitalisation duration; however, no significant differences were observed between the two groups (BBT group: 11.5 ± 2.0 days; BBT + E group: 12.6 ± 2.5 days; *p* = 0.410). We believe this outcome was strongly influenced by Japan's hospitalisation system, which is governed by the Diagnosis Procedure Combination (DPC) framework, whereby hospitalisation length is largely determined by the principal diagnosis. Under this system, variability in hospitalisation duration among patients tends to be minimal. Therefore, we excluded hospitalisation duration from the predefined outcome measures. Additionally, treatment procedures leading up to discharge were not standardised, which may have further contributed to this result. In settings where post–glucotoxicity treatment protocols are unified and discharge timing is determined based on clinical goals such as improvement in glycemic control, it is conceivable that the length of hospital stay may be shortened in the BBT + SGLT2 group.

## 5 Study Limitations

This study has several important limitations that should be acknowledged.

First, this was a single‐centre pilot study with a small number of participants, limiting the statistical power and generalisability of the results. Although all participants were deemed to require inpatient treatment, hospitalisation criteria may vary across institutions, and thus caution should be exercised when applying these findings to different healthcare systems. Additionally, all participants were Japanese, lacking racial and ethnic diversity. Future multicentre studies involving larger and more diverse populations are warranted.

Second, the observation period was limited to 7 days, which did not allow for evaluation of long‐term glycemic durability, β‐cell preservation or clinical outcomes. The short duration also did not allow for assessment of hospitalisation length or discharge‐related outcomes. Although a slight increase in ketone bodies was observed, its long‐term clinical relevance remains uncertain. While no cases of DKA occurred during the study, the risk cannot be completely ruled out; careful monitoring remains necessary.

Third, the open‐label design may have introduced observer bias, particularly regarding insulin dose adjustment and adverse event reporting. Although glucose and ketone levels were measured objectively, it is possible that clinicians were aware of treatment allocation during decision‐making.

Fourth, although no cases of DKA, ketosis or marked hyperglycemia were observed in this study, high‐risk populations—such as patients with a history of DKA, advanced renal dysfunction, or serious comorbidities—were excluded. Therefore, the findings may not be directly applicable to these vulnerable groups. Moreover, due to limited retrospective information, we could not fully assess participants' prior medication history, which may have influenced treatment responses. This study also did not systematically assess the adverse events associated with SGLT2 inhibitors, such as urinary tract or genital infections [36, 37], nor did it evaluate risks associated with long‐term use, leaving the safety profile incomplete.

Fifth, although the overall frequency of hypoglycemia was low in both groups, mild and asymptomatic nocturnal hypoglycemia was more frequently observed in the BBT + E group. Neither finger‐stick confirmation nor individualised insulin titration was performed, suggesting room for improvement in future protocol design.

Finally, future studies should assess the effects on pancreatic β‐cell function using indices such as C‐peptide levels, as well as evaluate patient‐reported outcomes including treatment satisfaction. Comparative trials with other antidiabetic agents, such as DPP‐4 inhibitors, as well as with conventional SIIT, could help clarify the therapeutic positioning and potential β‐cell protective effects of this approach.

## Author Contributions

Design: Nobutoshi Fushimi. Conduct/data collection: Nobutoshi Fushimi, Hiroki Hachiya, Tatsuya Iwasaka. Analysis: Nobutoshi Fushimi, Machi Nagao, Tomoki Masamura, Kohei Higashi. Writing manuscript: Nobutoshi Fushimi, Akihiro Mori.

## Ethics Statement

Approval of the research protocol: The study protocol was approved by the Ethics Committee of Ichinomiya Nishi Hospital. Approval date of Registry and the Registration No. of the study/trial: The study protocol was registered with the University Hospital Medical Information Network Clinical Trials Registry (UMIN000053224).

## Consent

All participants provided informed consent.

## Conflicts of Interest

The authors declare no conflicts of interest.

## Data Availability

Due to the nature of this research, the study participants did not agree for their data to be shared publicly; as such, supporting data is not available.
